# A solitary Peutz-Jeghers type polyp in the jejunum of a 19 year-old male

**DOI:** 10.1186/1757-1626-1-68

**Published:** 2008-07-31

**Authors:** Pieter PCJ ter Borg, Pieter PJ Westenend, Fried WLEM Hesp, Frans F van der Straaten, Wim W van de Vrie, Pieter P Honkoop

**Affiliations:** 1 Department of Gastroenterology, Albert Schweitzer Hospital, Dordrecht, The Netherlands; 2 Laboratory for Pathology Dordrecht, The Netherlands; 3 Department of Surgery, Albert Schweitzer Hospital, Dordrecht, The Netherlands; 4 Department of Radiology, Albert Schweitzer Hospital, Dordrecht, The Netherlands

## Abstract

A 19-year old male presented with melena and anemia. A duodenoscopy revealed no abnormalities, but a small bowel X-ray series demonstrated a large jejunal polyp. This 4 cm large polyp was visualised during peroperative small bowel endoscopy and was subsequently surgically removed. The polyp had the characteristic histologic appearance of a Peutz-Jeghers type polyp, but the patient had no other signs of Peutz-Jeghers syndrome, such as the characteristic mucocutaneous pigmentation, the presence of multiple polyps or a positive family history. After removal of the polyp, melena did not recur and his hemoglobin concentration normalized. Altogether, the patient does not fulfill the diagnostic criteria for Peutz-Jeghers syndrome and appears to have a solitary jejunal Peutz-Jeghers type polyp. All previously reported patients with such polyps were older than this patient.

## Background

Peutz-Jeghers syndrome (PJS) is characterized by the familial occurrence of gastrointestinal hamartomatous polyps in association with mucocutaneous hyperpigmentation. The diagnosis of PJS can be made using the WHO criteria: [1] 3 or more histologically confirmed Peutz-Jeghers type polyps (PJP), or, [2] any number of PJP with a family history of PJS, or, [3] characteristic, prominent, mucocutaneous pigmentation with a family history of PJS, or, [4] any number of PJP and characteristic, prominent mucocutaneous pigmentation. Patients with PJS are at increased risk of both gastrointestinal and extraintestinal malignancies. The gastrointestinal hamartomatous polyps in patients with PJS have a distinct histological appearance with interdigitating smooth muscle fibers forming a characteristic branching tree pattern (arborization) [1]. These polyps usually occur in the context of the PJS. However, several case reports and small series of patients with a solitary PJP, but without affected family members or the typical mucocutaneous findings observed in PJS, have been published [2-15]. With the exception of a 22-year old female, all reported patients with such solitary PJP's were over 40 years of age. We describe a 19 year-old male patient presenting with melena as a result of bleeding from a large PJP in the jejunum.

## Case presentation

**Figure 1:**
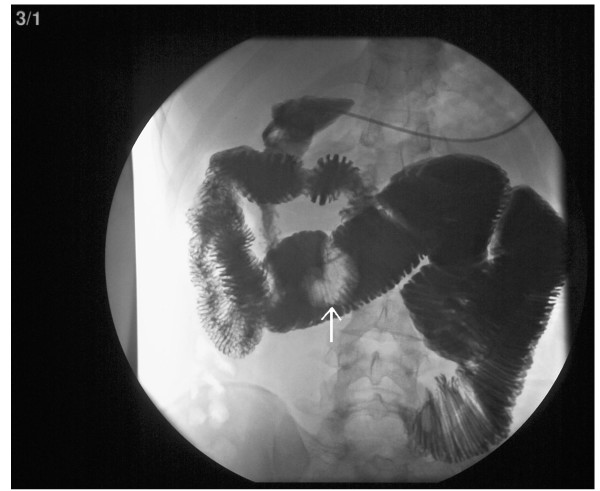
Small bowel series demonstrating a large jejunal polyp.

A 19-year old male Caucasian patient visited the emergency department of our hospital because of melena. He was previously healthy and used no medication but had noticed black stools one week before his visit. There had been no abdominal discomfort, nausea or vomiting. None of his family members had gastrointestinal diseases, and he used no alcohol, did not smoke or use elicit drugs. His occupation is unknown. On physical examination the abdomen was nontender and without palpable masses. Rectal examination revealed black stools. His weight was 62 kg. and his length 1,87 m. Laboratory tests showed an iron deficiency anemia with a hemoglobin concentration of 4.7 mmol/L (normal > 8,5 mmol/L) and a serum ferritin concentration of 4 μg/L (normal > 30 μg/L). Liver function tests, serum creatinine, albumin, CRP and glucose were all normal. Duodenoscopy showed a normal esophageal, gastric and duodenal mucosa without evidence of recent bleeding. A Meckel scan showed no evidence for a Meckel's diverticulum. A small bowel series however, demonstrated a large polyp in the proximal jejunum and possibly a smaller polyp in the distal jejunum (Figure 1).

**Figure 2:**
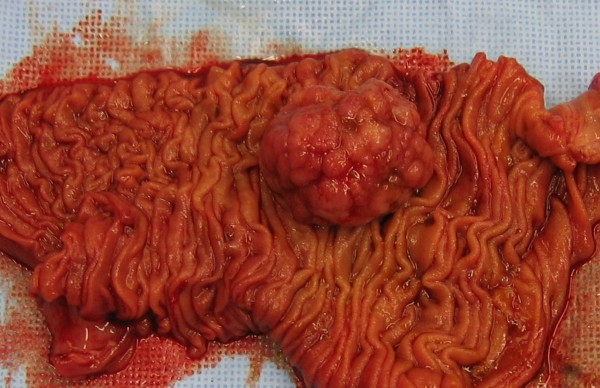
Resected jejunal segment including the 40 mm large polyp.

As this polyp was the probable cause of the patient's bleeding, a laparotomy with peroperative small bowel endoscopy was performed. Approximately 50 cm beyond Treitz' ligament, a multilobular, 40 mm large polyp was seen, which was subsequently surgically resected (Figure 2). There were no other small bowel polyps; the suspected smaller polyp seen on the small bowel series was therefore most likely an artefact.

**Figure 3:**
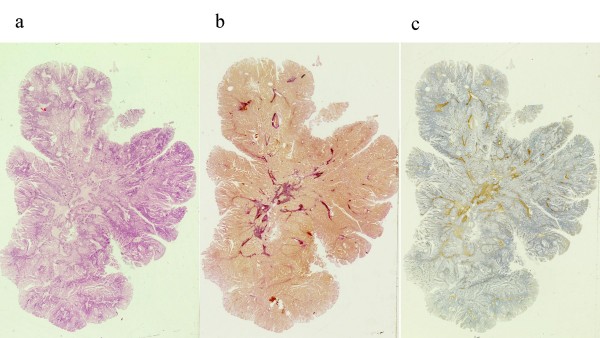
**Microscopic images of the polyp**. A. Haematoxylin eosin staining. B. Van Gieson staining. C. Immunostaining for smooth muscle actin.

Pathologic examination of this polyp demonstrated a hamartomatous polyp with arborization of smooth muscle compatible with a PJP (Figure 3). No signs of dysplasia were seen and the surrounding tissue had a normal histological appearance. Immunohistochemistry was negative for p53, focally positive for cyclin D1 and the basal parts of the cryps were positive for Ki67/MIB1.

The postoperative course has been unremarkable and no further gastrointestinal bleeding has occurred. Total colonoscopy did not show any additional polyps. A repeated, extensive family history, performed by a clinical genetics consultant, did not reveal clues (including pigmentation, nasal or gastrointestinal polyps or typical secondary tumors) to the occurrence of PJS in family members.

## Discussion

Our patient presented with upper gastrointestinal bleeding from a PJP. The presenting gastrointestinal symptom in a series of patients with PJS was bleeding in 14%, obstruction in 43%, pain in 23% and extrusion of a rectal polyp in 7% of patients [16].

PJS is an autosomal dominant hamartomatous polyposis syndrome which is associated with mucocutaneous hyperpigmentation. The hamartoma's in PJS are histologically characterized by smooth muscle fibers showing a typical branching pattern. However, several patients with PJP's without other characteristics of PJS have previously been described [2-15]. Because of the absence of involved family members, the lack of mucocutaneous pigmentation characteristic of PJS and the presence of a solitary polyp, a solitary PJP might be a disease entity distinct from PJS. There is, however, controversy about the occurrence of solitary PJP's. In most case reports and series, the clinical and histological criteria were not fully documented and there was usually no extended follow-up [17]. In a study on the occurrence of PJP's in a large teaching hospital including extensive review of clinical and histologic data, only 3 patients with solitary PJP's were identified and all these patients had other conditions suggestive of PJS (one with pancreatic cancer and extensive family history of GI malignancies, one with pancreatic cancer and one with bilateral ovarian masses and a glomus tympanicum tumor). The authors suggest that patients with solitary PJP's might have an incomplete or early form of PJS [17]. In addition, mucocutaneous pigmentation, which may be the only manifestation of a patient with PJS, decreases with age and family members might therefore have clinically occult, undetected PJP's without mucocutaneous pigmentation.

Of the patients reported with solitary PJP's, most were over 40 years of age, which is considerably older than the usual age at presentation of patients with PJS. In a recent case series of 8 patients with solitary PJP's, the mean age at diagnosis was 56 years and the youngest patient was 33 years old [12]. In 2 of these 8 patients, the polyp was localized in the duodenum, in the remaining 6 patients it was found in the stomach or colon. The largest polyp had a size of 25 mm, compared to 40 mm in our patient.

Patients with PJS are at increased risk of developing both intestinal and extraintestinal malignancies. Several rare disorders, such as Sertoli cell tumor of the testis and sex cord tumors with annular tubules occur relatively frequently in patients with PJS [1]. Although no occurrences of these rare extraintestinal tumors have been reported in patients with solitary PJP's, two patients in one study presented with a simultaneous pancreatic carcinoma, whereas in another study 1 of 8 patients died of colon cancer [17].

In 50–94% of patients with PJS, a mutation of the LKB1/STK11 gene is found. This gene codes for a serine threonine kinase. Although the exact role of this protein kinase is unknown, it might function as a tumor suppressor. In addition, it seems to play a role in regulating cell polarity, and might thus be the cause of the development of hamartomatous polyps. However, in a significant proportion of families mutations in this gene have not been found, suggesting that other genes responsible for the development of PJS exist. In only one of the published cases of solitary PJP a LKB1/STK11 mutation analysis was performed; a mutation was not found [8]. It remains therefore unknown whether these mutations also occur in patients with a solitary PJP.

## Conclusion

We described a patient with a large, bleeding solitary PJP in the jejunum at an age typical of patients with PJS, but considerably younger than previously reported patients with solitary PJP's.

## Abbreviations

PJP: Peutz-Jeghers type polyp; PJS: Peutz-Jeghers syndrome; CRP: C-reactive Protein.

## Competing interests

The authors declare that they have no competing interests.

## Authors' contributions

PB reviewed the case and relevant literature and prepared and revised the manuscript. PW, PH, WV, WH and FS were involved in the patient care and reviewed and edited the manuscript. PH and WV were involved clinical and outpatient patient care, WH performed the surgery, FS performed/reviewed all imaging studies and PW performed the histological examination. All authors read and approved the final manuscript.

## Consent

Written informed consent was obtained from the patient for publication of this case report and accompanying images. A copy of the written consent is available for review by the Editor-in-Chief of this journal.

## References

[B1] Schreibman IR, Baker M, Amos C, McGarrity TJ (2005). The hamartomatous polyposis syndromes: a clinical and molecular review. Am J Gastroenterol.

[B2] Acea Nebril B, Taboada Filgueira L, Parajo Calvo A, Gayoso Garcia R, Gomez Rodriguez D, Sanchez Gonzalez F, Sogo Manzano C (1993). Solitary hamartomatous duodenal polyp; a different entity: report of a case and review of the literature. Surg Today.

[B3] Bott SJ, Hanks JB, Stone DD (1986). Solitary hamartomatous polyp of the duodenum in the absence of familial polyposis. Am J Gastroenterol.

[B4] Grisendi A, Lonardo A (1990). Solitary Peutz-Jeghers type polyp of the stomach. Endoscopy.

[B5] Hanna RM, Dahniya MH, Seddiq MA, Kamel H (1994). Case report: a case of solitary Peutz-Jegher's hamartoma in the small bowel with angiographic evaluation. Br J Radiol.

[B6] Hunt J, Tindal D (1996). Solitary gastric Peutz-Jeghers polyp and angiolipoma presenting as acute haemorrhage. Aust N Z J Surg.

[B7] Ichiyoshi Y, Yao T, Nagasaki S, Sugimachi K (1996). Solitary Peutz-Jeghers type polyp of the duodenum containing a focus of adenocarcinoma. Ital J Gastroenterol.

[B8] Kitaoka F, Shiogama T, Mizutani A, Tsurunaga Y, Fukui H, Higami Y, Shimokawa I, Taguchi T, Kanematsu T (2004). A solitary Peutz-Jeghers-type hamartomatous polyp in the duodenum. A case report including results of mutation analysis. Digestion.

[B9] Kuwano H, Takano H, Sugimachi K (1989). Solitary Peutz-Jeghers type polyp of the stomach in the absence of familial polyposis coli in a teenage boy. Endoscopy.

[B10] Naitoh H, Sumiyoshi Y, Kumashiro R, Inutsuka S, Fujita K, Yamamoto T, Murayama H (1988). A solitary Peutz-Jeghers type hamartomatous polyp in the duodenum--a case report. Jpn J Surg.

[B11] Nakayama H, Fujii M, Kimura A, Kajihara H (1996). A solitary Peutz-Jeghers-type hamartomatous polyp of the rectum: report of a case and review of the literature. Jpn J Clin Oncol.

[B12] Oncel M, Remzi FH, Church JM, Goldblum JR, Zutshi M, Fazio VW (2003). Course and follow-up of solitary Peutz-Jeghers polyps: a case series. Int J Colorectal Dis.

[B13] Sakadamis AK, Ballas KD, Fardellas JG, Papanikolaou A (2001). A solitary gastric Peutz-Jeghers type polyp: report of a case. Surg Today.

[B14] Sone Y, Nakano S, Takeda I, Kumada T, Kiriyama S, Hisanaga Y (2000). Solitary hamartomatous polyp of Peutz-Jeghers type in the jejunum resected endoscopically. Gastrointest Endosc.

[B15] Suzuki S, Hirasaki S, Ikeda F, Yumoto E, Yamane H, Matsubara M (2008). Three cases of Solitary Peutz-Jeghers-type hamartomatous polyp in the duodenum. World J Gastroenterol.

[B16] Utsunomiya J, Gocho H, Miyanaga T, Hamaguchi E, Kashimure A (1975). Peutz-Jeghers syndrome: its natural course and management. Johns Hopkins Med J.

[B17] Burkart AL, Sheridan T, Lewin M, Fenton H, Ali NJ, Montgomery E (2007). Do sporadic Peutz-Jeghers polyps exist? Experience of a large teaching hospital. Am J Surg Pathol.

